# Molecular identification and distribution of *Dictyocaulus* spp. in wild ungulates in Lithuania

**DOI:** 10.1017/S0031182026102704

**Published:** 2026-08

**Authors:** Silvija Misailovė-Ribokė, Indrė Lipatova, Irma Ražanskė, Povilas Sakalauskas, Vytautas Mažeika, Muza Kirjušina, Artūras Kibiša, Algimantas Paulauskas

**Affiliations:** 1 Faculty of Natural Sciences, Vytautas Magnus University, Kaunas, Lithuania; 2 Institute of Life Sciences and Technology, Daugavpils Universitate, Daugavpils, Latvia

**Keywords:** 18S rDNA, *Alces alces*, *Bison bonasus*, *Capreolus capreolus*, *Cervus elaphus*, *cox1*, *Dictyocaulus* species, lungworm, phylogeny, prevalence

## Abstract

This study aimed to investigate the diversity, prevalence and host specificity of *Dictyocaulus* lungworms in free-ranging wild ungulates in Lithuania using morphological and molecular approaches. A total of 129 individuals representing 4 host species (red deer, roe deer, moose and European bison) were examined. Four *Dictyocaulus* species were identified: *D. cervi, D. skrjabini, D. capreolus* and *D. viviparus bisontis*. Infection parameters varied markedly among host species. Red deer showed the highest prevalence and parasite diversity, whereas European bison exhibited low prevalence but relatively high infection intensity. Moose displayed intermediate infection levels with a high proportion of co-infections, while roe deer were infected exclusively with the host-specific species *D. capreolus*. Co-infections involving multiple *Dictyocaulus* species were common in cervids, indicating complex transmission dynamics. Molecular analyses based on mitochondrial (*cox1*) and nuclear (*SSU rDNA*) markers confirmed species identification and revealed clear genetic differentiation among taxa. Phylogenetic incongruences between markers suggest potentially complex evolutionary relationships, possibly influenced by co-infections and host overlap. These findings provide the first comprehensive insight into *Dictyocaulus* infections in wild ungulates in Lithuania and highlight the importance of combining morphological and molecular methods for accurate parasite identification. The results contribute to a better understanding of lungworm epidemiology and host–parasite interactions in wildlife and emphasize the need for improved surveillance strategies.

## Graphical abstract

**Figure:**
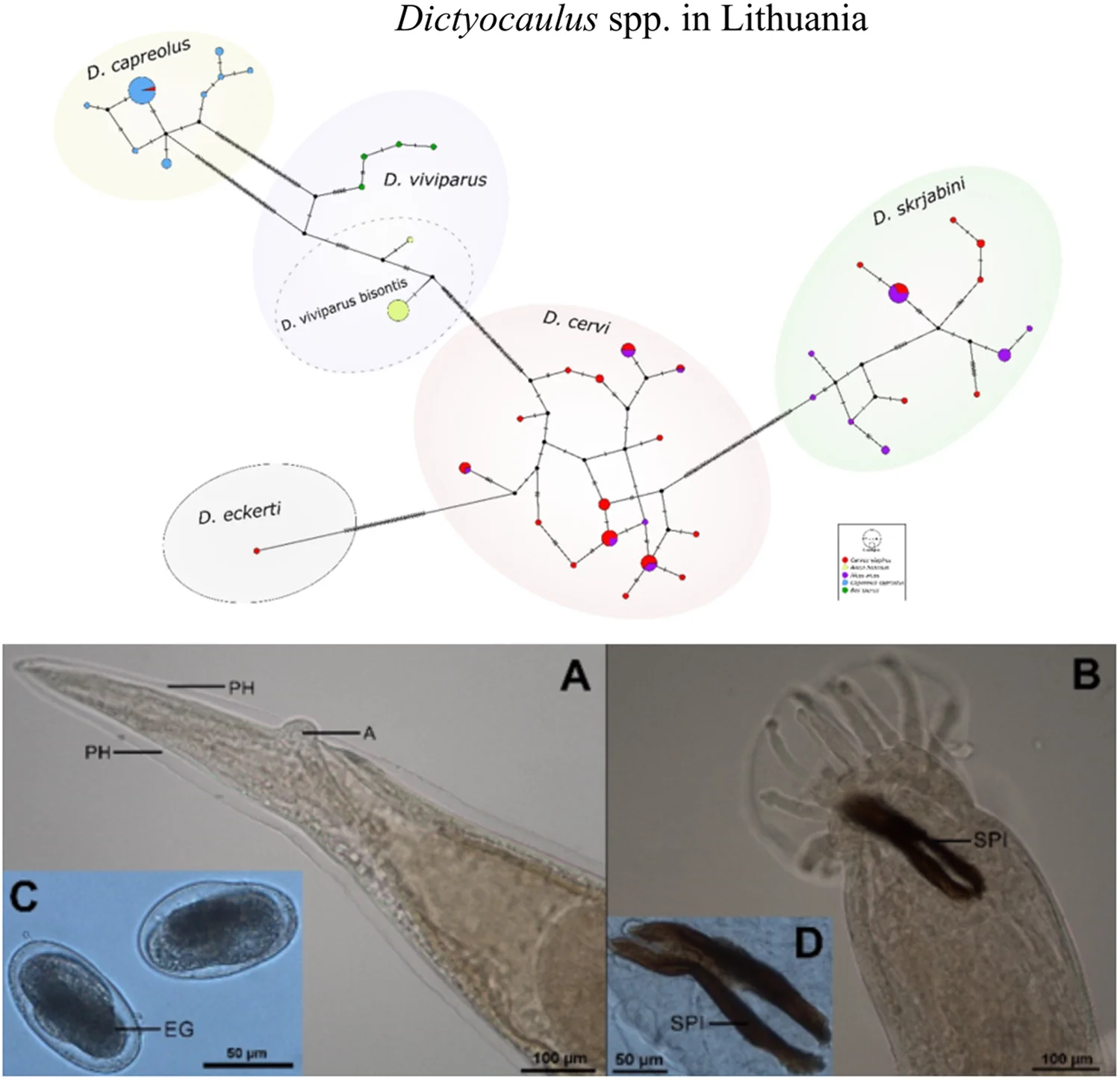

## Introduction

*Dictyocaulus* species are lungworms that occur in wild and domestic ungulates across many parts of the world. These lung nematodes cause dictyocaulosis, a parasitic pneumonia and bronchitis (Panuska, 2006; Pyziel et al., 2017). The number of worms in the lungs affects the severity of the infection. While a small number of worms may cause no noticeable symptoms, a large infestation can be fatal (Panuska, 2006; Cârstolovean et al., 2024). Morphological characteristics alone are insufficient for species-level identification of *Dictyocaulus* parasites. Prior to 2016, 6 *Dictyocaulus* species had been described: *Dictyocaulus viviparus, Dictyocaulus filaria, Dictyocaulus arnfieldi, Dictyocaulus cameli, Dictyocaulus eckerti* and *Dictyocaulus capreolus*. Since 2017, 2 additional species, *Dictyocaulus cervi* and *Dictyocaulus skrjabini*, have been described using combined morphological and molecular identification methods (Pyziel et al., 2017, 2023). Accurate identification of *Dictyocaulus* species is essential not only for clarifying their taxonomy but also for understanding host specificity, geographical distribution, potential cross-transmission among different host species (e.g. between cervids and bovines), and implementing effective control strategies for dictyocaulosis (Gasser et al., 2012).

In Lithuania, 4 species of native wild ungulates are free-ranging: moose (*Alces alces*), roe deer (*Capreolus capreolus*), red deer (*Cervus elaphus*) and European bison (*Bison bonasus*). As the populations of these animals grow and increasingly share grazing areas with domestic ruminants, they may act as reservoirs of *Dictyocaulus* lungworms and facilitate parasite transmission between wildlife and livestock (Cafiso et al., 2023; González-velo et al., 2025). In Europe, several *Dictyocaulus* species have been reported in wild and domestic ruminants, including *D. cervi*, which is commonly associated with cervids, and *D. viviparus*, which primarily infects cattle but has been detected in wildlife (Ploeger, 2002; Höglund et al., 2006; González-velo et al., 2025). However, the distribution and host range of these species may vary geographically, and their occurrence in wild ungulates is still insufficiently characterized in many regions.

Available data on *Dictyocaulus* spp. in Lithuania remain scarce, and to date no comprehensive studies have investigated their diversity and distribution. Early studies on the helminth fauna of roe deer in Lithuania reported a low prevalence of *Dictyocaulus* species; however, species-level identification remains uncertain and no detailed information on their geographical distribution was provided (Kazlauskas and Pužauskas, 1974). Therefore, the diversity, host associations and distribution of these parasites in Lithuanian wildlife are still poorly understood, and their molecular characterization has not been sufficiently investigated. The aim of this study was to determine the distribution and species composition of *Dictyocaulus* parasites infecting different wild ungulate hosts using genetic markers for species-level identification.

## Materials and methods

### Sample collection and morphological identification

Between 2021 and 2024, lower respiratory tracts (trachea and lungs) were collected from a total of 41 red deer, 33 roe deer, 14 moose and 41 European bison in Lithuania. Red deer were legally hunted in 4 municipalities: Akmenė (*n* = 1), Jurbarkas (*n* = 1), Raseiniai (*n* = 3) and Kėdainiai (*n* = 36); roe deer in 2 municipalities: Jurbarkas (*n* = 5) and Kėdainiai (*n* = 28); and moose in 1 municipality: Kėdainiai (*n* = 14). In accordance with the European bison protection plan, sampling was conducted with permission from the Environmental Protection Department under the Ministry of Environment of the Republic of Lithuania. Samples were obtained only from individuals that had died naturally, were killed in road traffic accidents, or were removed from the population for health-related reasons as part of an approved management plan. These samples were collected in 2 municipalities: Kėdainiai (*n* = 28) and Varėna (*n* = 3) (Supplementary Fig. 1).

Tracheas, bronchi and bronchioles were examined for the presence of nematodes by dissecting and flushing. The fluid was collected and sediment was examined under an SMZ-171-BLED stereomicroscope (Motic, Hong Kong). The adult nematodes were isolated, briefly washed in water solution for any host debris removal, and stored in 70 % ethanol at +4 °C for further morphological and molecular investigation. Primary identification of nematode species was done by morphological criteria (Divina et al., 2000; Pyziel et al., 2017, 2023; Jurankova et al., 2019). The species representing the figures were taken and processed by coauthors at Daugavpils University using Nikon Eclipse 90i microscope equipped with Nikon DS-5MC camera 5.0 M and NIS-Elements Advanced Research software. Parasitological indices, including parasite prevalence, mean intensity and mean abundance, were calculated according to Bush et al. (1997).

### DNA extraction, polymerase chain reaction and sequencing

Genomic DNA from adult lungworms was extracted using a QIAamp DNA Mini Kit (QIAGEN, Germany), following the manufacturer’s protocol. Molecular identification of lungworm species was performed by conventional polymerase chain reaction (PCR) assays targeting 2 genetic markers: a 658 bp fragment of the mitochondrial cytochrome c oxidase subunit I gene (*cox1*) and an approximately 1200 bp fragment of the *18S* small subunit ribosomal DNA (*SSU rDNA*) gene (Table 1), as previously described (Folmer et al., 1994; Pyziel et al., 2017).

**Table 1.** PCR target genes and primers used in this study for identification of *Dictyocaulus* spp Table 1 long description.

Target gene	Primer	Oligonucleotide sequences (5′-3′)	Reference
*cox1*	LCO1490	5′-GGTCAACAAATCATAAAGATATTGG-3′	Folmer et al., 1994
	HCO2198	5′-TAAACTTCAGGGTGACCAAAAAATCA-3′	
*SSU rDNA*	NF50	5′-TGAAATGGGAACGGCTCAT-3′	Pyziel et al., 2017
	BNR1	5′-ACCTACAGATACCTTGTTACGAC-3′	

PCR amplification of the *cox1* gene was carried out in a total volume of 20 μL, containing 4 μL of DNA template, 10 μL of Dreamtaq Green PCR Master Mix (Thermo Fisher Scientific, Vilnius, Lithuania) and 1.7 μL of 10 mM forward and reverse primers (Table 1). The following conditions were used for PCR amplification: an initial denaturation at 94°C for 1 min, followed by 5 cycles of 94°C for 60 s, 45°C for 90 s and 72°C for 90 s, then 35 cycles of 94°C for 60 s, 50°C for 90 s and 72°C for 60 s, with a final extension at 72°C for 5 min (Folmer et al., 1994).

Amplification of the *SSU rDNA* fragment was also performed in a total volume of 20 μL, consisting of 4 μL of DNA template, 10 μL of Dreamtaq Green PCR Master Mix (Thermo Fisher Scientific, Vilnius, Lithuania) and 2 μL of 10 mM each primer (Table 1). The PCR thermal profile included an initial denaturation at 95°C for 5 min, followed by 40 cycles of 94°C for 30 s, 55°C for 30 s and 72°C for 30 s, with a final extension at 72°C for 5 min (Pyziel et al., 2017). For each PCR run, molecular-grade nuclease-free water was used as a negative control.

PCR products of good quality were extracted from the 1.5% agarose gel and purified with a GeneJET Gel Extraction Kit (Thermo Fisher Scientific, Lithuania) according to the manufacturer’s instructions and sequenced (Macrogen, the Netherlands). The obtained sequences were edited, aligned with one other and compared with the gene sequence data available from GenBank, using the MEGA X software package (Kumar et al., 2018). Phylogenetic trees were constructed using the maximum-likelihood method with the Tamura–Nei model. Bootstrap support was calculated by means of 1000 replicates. A *cox1* haplotype network of the *Dictyocaulus* spp. was generated using the TCS method implemented in PopART v1.7 (Leigh and Bryant, 2015).

The obtained *cox1* gene sequences for representative samples were deposited in the GenBank database under the following accession numbers: PV176498 – PV176509 for *D. skrjabini*, PV176479 – PV176497 for *D. cervi*, PV176475 – PV176478 and PV523589 for *D. capreolus* and PV444367 – PV444371 for *D. viviparus bisontis*. The *SSU rDNA* sequences were also submitted to GenBank and are available under the following accession numbers: PV195231 – PV195242 for *D. skrjabini*, PV195243 – PV195251 for *D. cervi*, PV195252 – PV195261 for *D. capreolus* and PV654200 – PV654203 for *D. viviparus bisontis*.

## Results

Parasitological examination revealed the presence of *Dictyocaulus* spp. in 78.1% of red deer (32/41), 21.2% of roe deer (7/33), 57.1% of moose (8/14) and 12.2% of European bison (5/41). Infections were detected across all sampling sites, with prevalence varying among host species and locations (Supplement Table 1).

**Figure 1.**
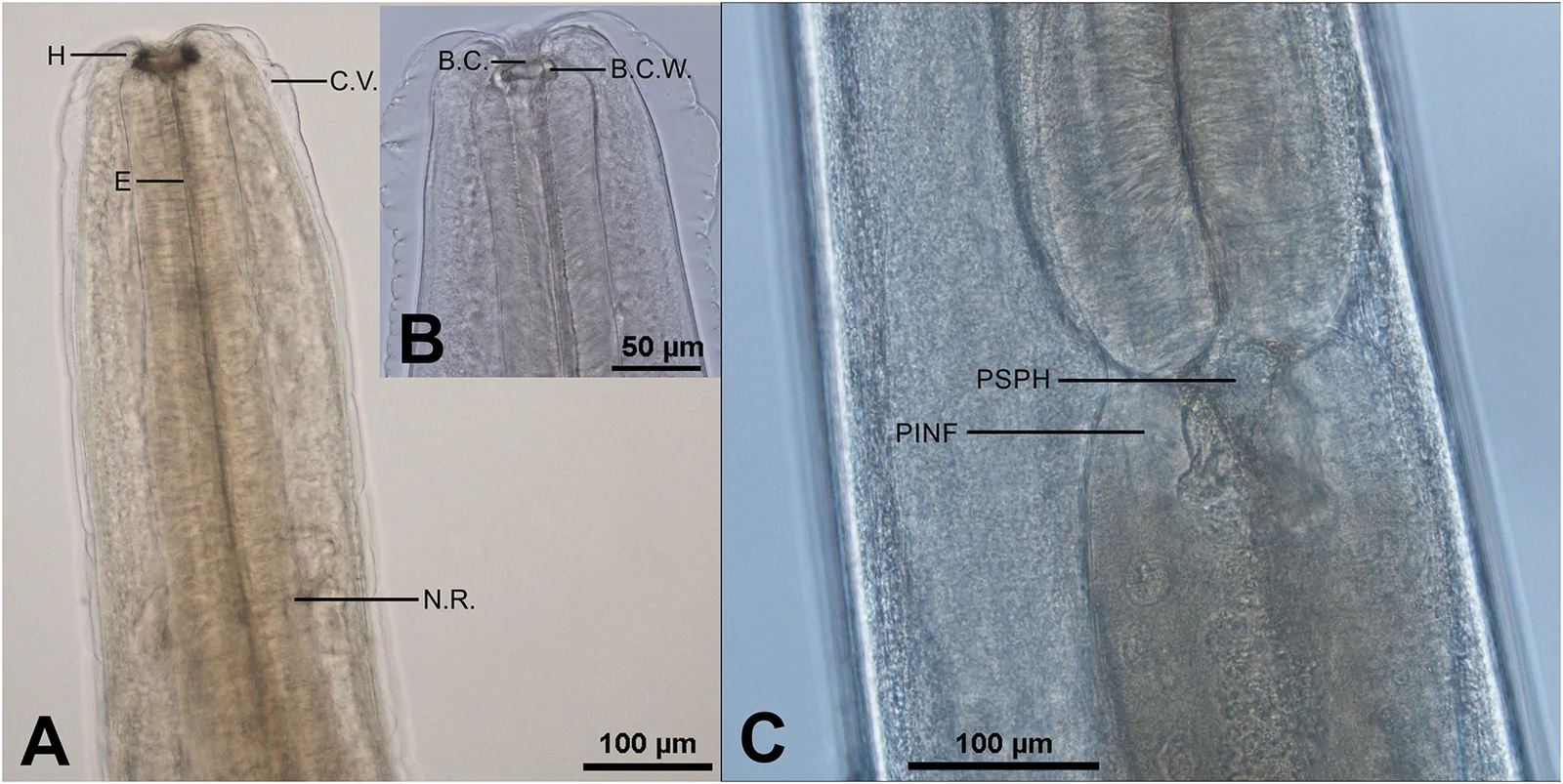
*Dictyocaulus cervi.* (A) Female anterior end in optical section showing H, head; C.V., cephalic vesicle; E, oesophagus; N.R., nerve ring. (B) Female anterior end in optical section showing B.C., Buccal capsule; B.C.W., Buccal capsule wall. (C) Female middle part in optical section showing PSPH, posterior sphincter; PINF, posterior infundibulum.

**Figure 2.**
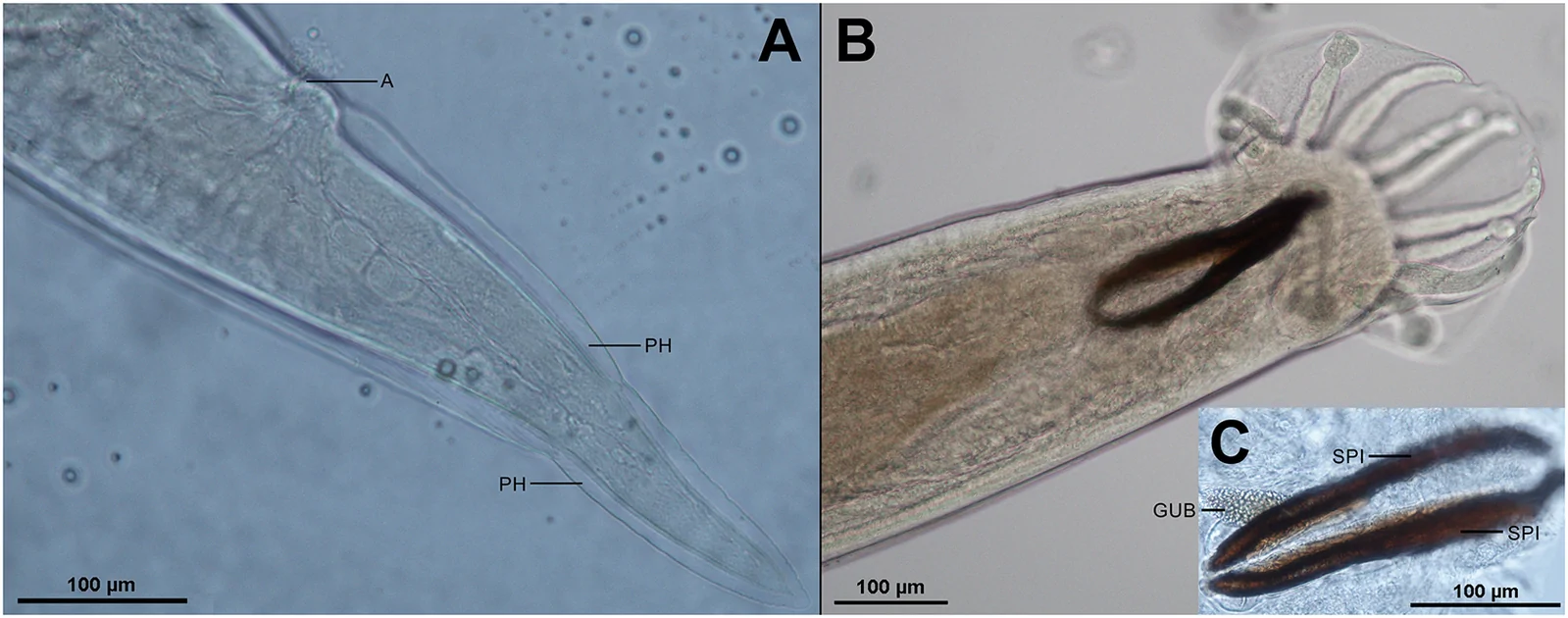
*Dictyocaulus cervi.* (A) Female tail in optical section showing A, anus; PH, phasmids. (B and C) Male genital system in optical section showing GUB, gubernaculum; SPI, spicules.

**Figure 3.**
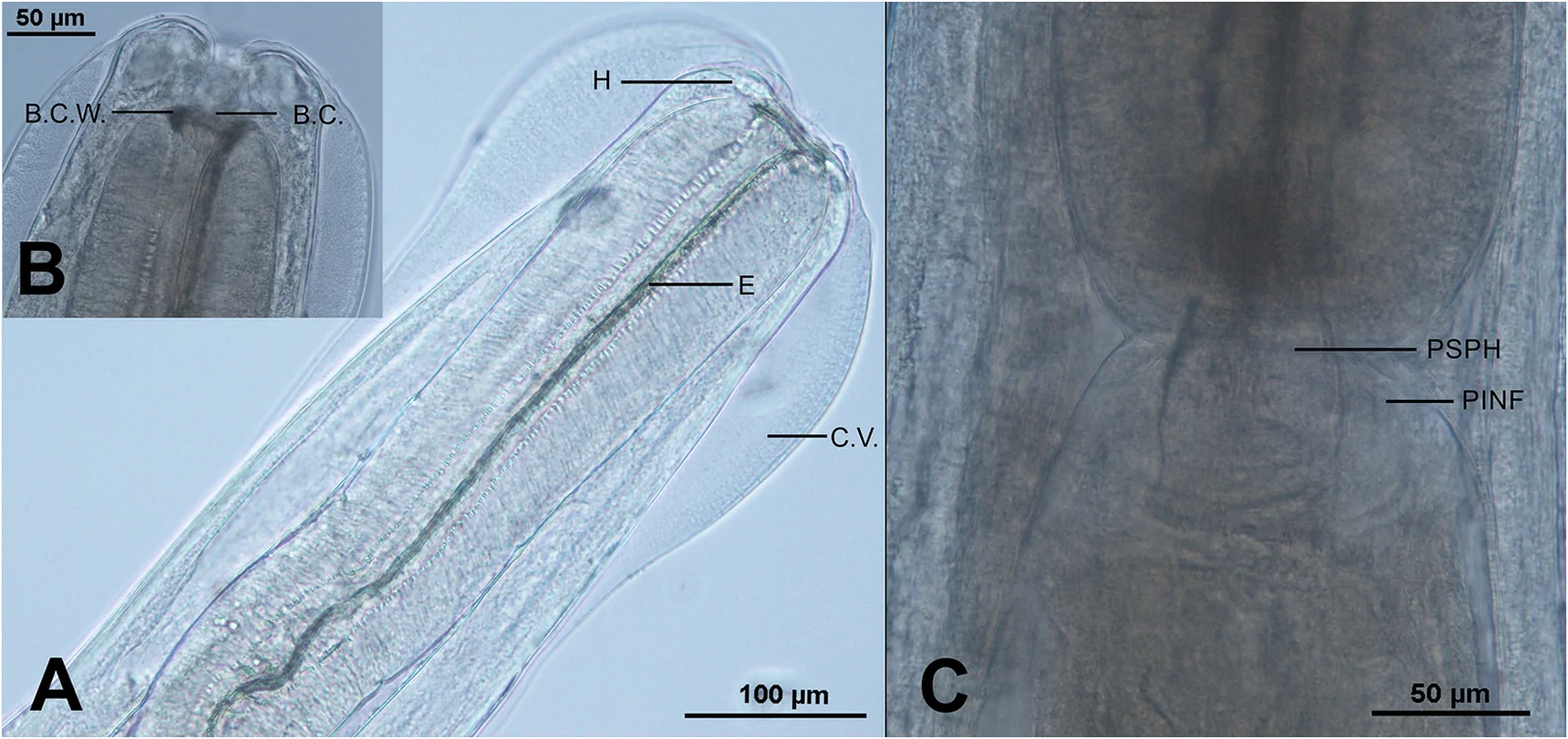
*Dictyocaulus skrjabini.* (A) Female anterior end in optical section showing H, head; C.V., cephalic vesicle; E, oesophagus. (B) Female anterior end in optical section showing B.C., Buccal capsule; B.C.W., Buccal capsule wall. (C) Female middle part in optical section showing PSPH, posterior sphincter; PINF, posterior infundibulum.

**Figure 4.**
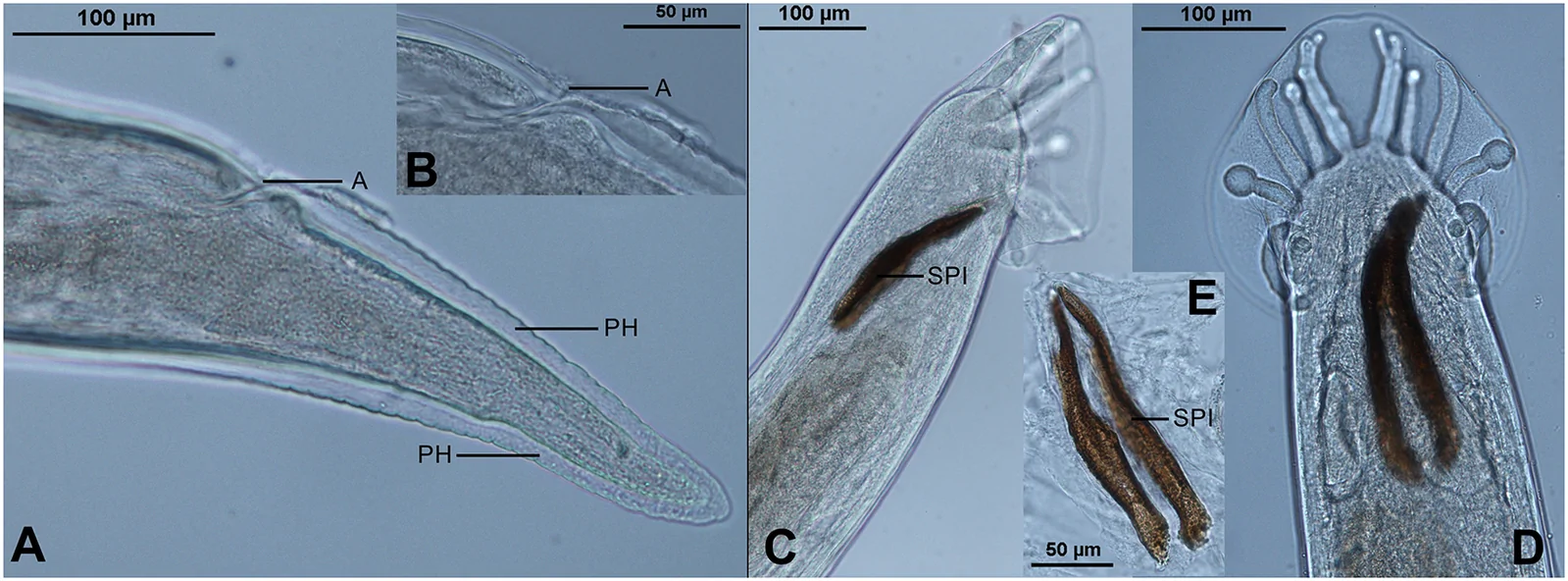
*Dictyocaulus skrjabini.* (A and B) Female tail in optical section showing A, anus; PH, phasmids. (C–E) Male genital system in optical section showing SPI, spicules.

**Figure 5.**
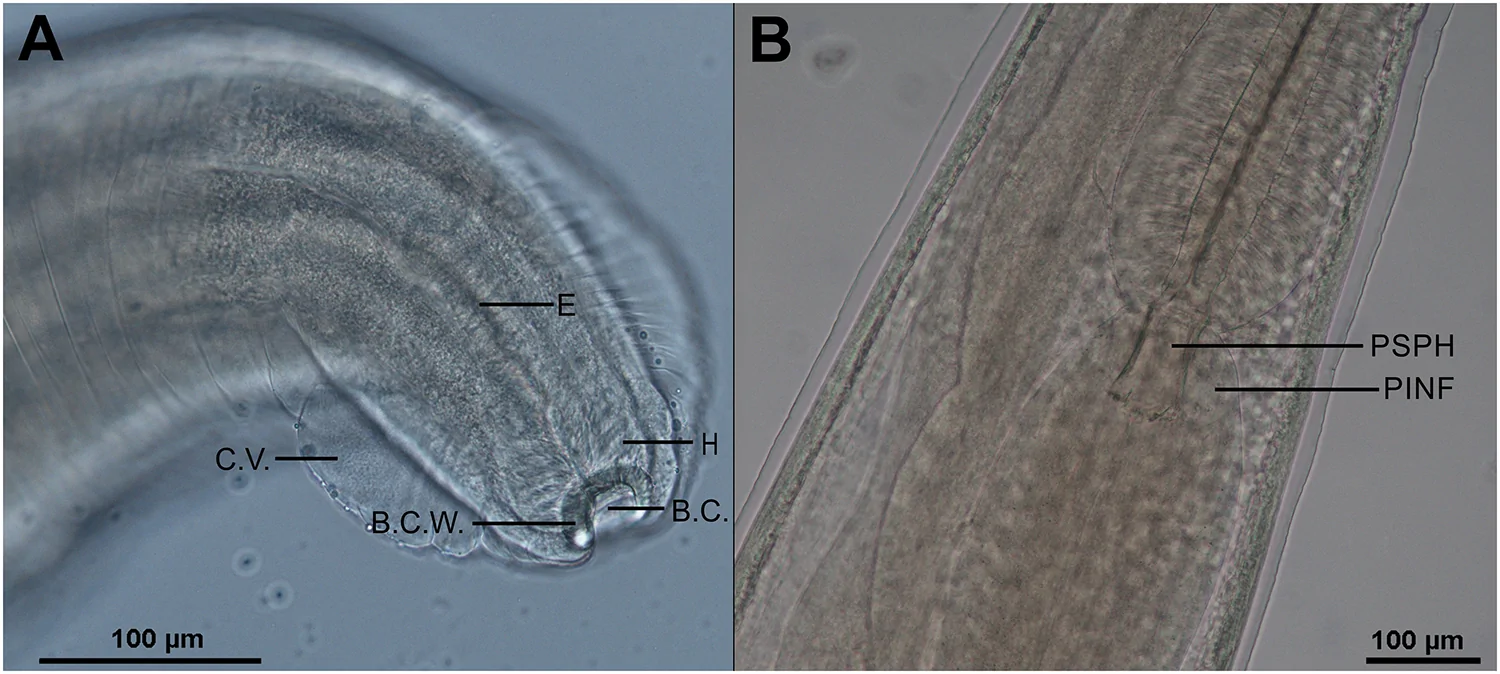
*Dictyocaulus capreolus.* (A) Female anterior end in optical section showing H, head; C.V., cephalic vesicle; E, oesophagus; B.C., Buccal capsule; B.C.W., Buccal capsule wall. (B) Female middle part in optical section showing PSPH, posterior sphincter; PINF, posterior infundibulum.

**Figure 6.**
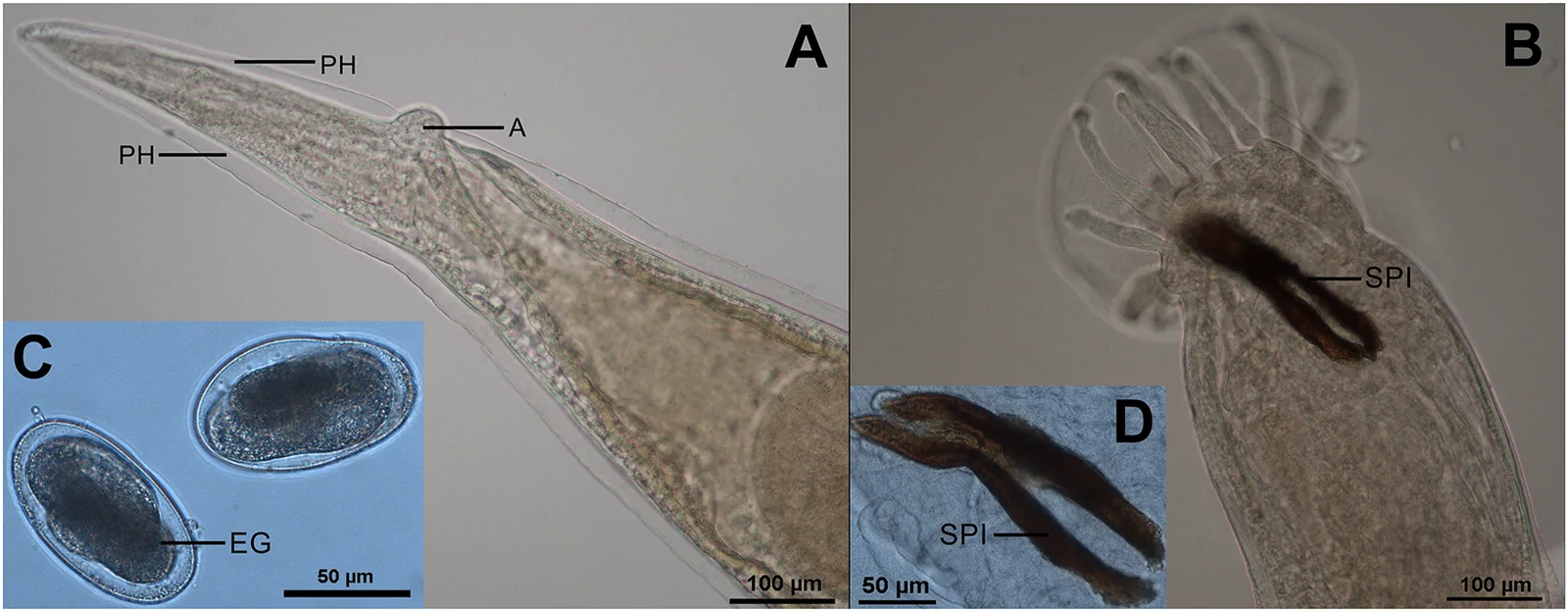
*Dictyocaulus capreolus.* (A) Female tail in optical section showing A, anus; PH, phasmids. (B and D) Male genital system in optical section showing SPI, spicules; (C) showing EG, eggs.

Based on *cox1* and *SSU rDNA* sequences, 4 *Dictyocaulus* species were identified: *D. capreolus, D. cervi, D. skrjabini* and *D. viviparus* subsp. *bisontis*. Representative individuals of each molecularly confirmed species were photographed for visual documentation (Figures 1–6). Species assignment based on 2 genetic markers yielded identical results, with no discrepancies observed between *cox1* and *SSU rDNA*.

Infection parameters varied among host species and *Dictyocaulus* species (Table 2). The highest prevalence and mean abundance were recorded for *D. cervi* in red deer (*C. elaphus*), followed by *D. skrjabini*, whereas *D. capreolus* occurred only sporadically in this host. In roe deer (*C. capreolus*), only *D. capreolus* was detected. Moose (*A. alces*) harboured both *D. cervi* and *D. skrjabini*, with similar prevalence and mean abundance values. In European bison (*B. bonasus*), only *D. viviparus bisontis* was detected.

**Table 2.** Infection of ungulates by lungworms in Lithuania Table 2 long description.

Host	*N*	*n*	*Dictyocaulus* spp.	*P* (95% CI)	MA	*I*_min_–*I*_max_
*C. elaphus*	41	27	*D. cervi*	65.9% (50.5–78.4)	22.24	1–197
		12	*D. skrjabini*	29.3% (17.6–44.5)	2.73	1–45
		1	*D. capreolus*	2.4% (0.4–12.6)	0.27	11–11
*C. capreolus*	33	7	*D. capreolus*	21.2% (10.5–37.0)	0.88	1–9
*A. alces*	14	5	*D. cervi*	35.7% (16.3–61.2)	1.79	1–10
		6	*D. skrjabini*	42.9% (21.4–67.4)	1.71	2–11
*B. bonasus*	41	5	*D. viviparus bisontis*	12.2% (5.3–25.3)	2.51	5–57

*N*, number of hosts studied; *n*, number of host infected; *P* (95%CI), prevalence with 95% confidence interval; MA, mean abundance; *I*_min_–*I*_max_, infection intensity range.

Co-infections with multiple *Dictyocaulus* species were observed in red deer and moose (Table 3). In red deer, most co-infections involved *D. cervi* and *D. skrjabini*, while 1 animal harboured 3 species: *D. capreolus, D. cervi* and *D. skrjabini*. In moose, co-infections involved *D. cervi* and *D. skrjabini*. No co-infections were recorded in roe deer or European bison.

**Table 3.** *Dictyocaulus* spp. numbers in different hosts, hosts with co-infections Table 3 long description.

	Total number of lungworms									
	*D. capreolus*		*D. cervi*		*D. skrjabini*		*D. viviparus bisontis*		Hosts with co-infection/number of infected hosts	
Host species	♂	♀	♂	♀	♂	♀	♂	♀	D.ce + D.s	D.ca + D.ce + D.sk^a^
Red deer	4	7	321	591	54	58	–		6/32	1/32
Roe deer	10	19	–	–	–	–	–	–	–	–
Moose	–		7	18	9	15	–		4/8	–
European Bison	–		–		–		37	66	–	–

a D.ca, *D. capreolus*; D.ce, *D. cervi*; D.sk, *D. skrjabini.*

**Figure 7.**
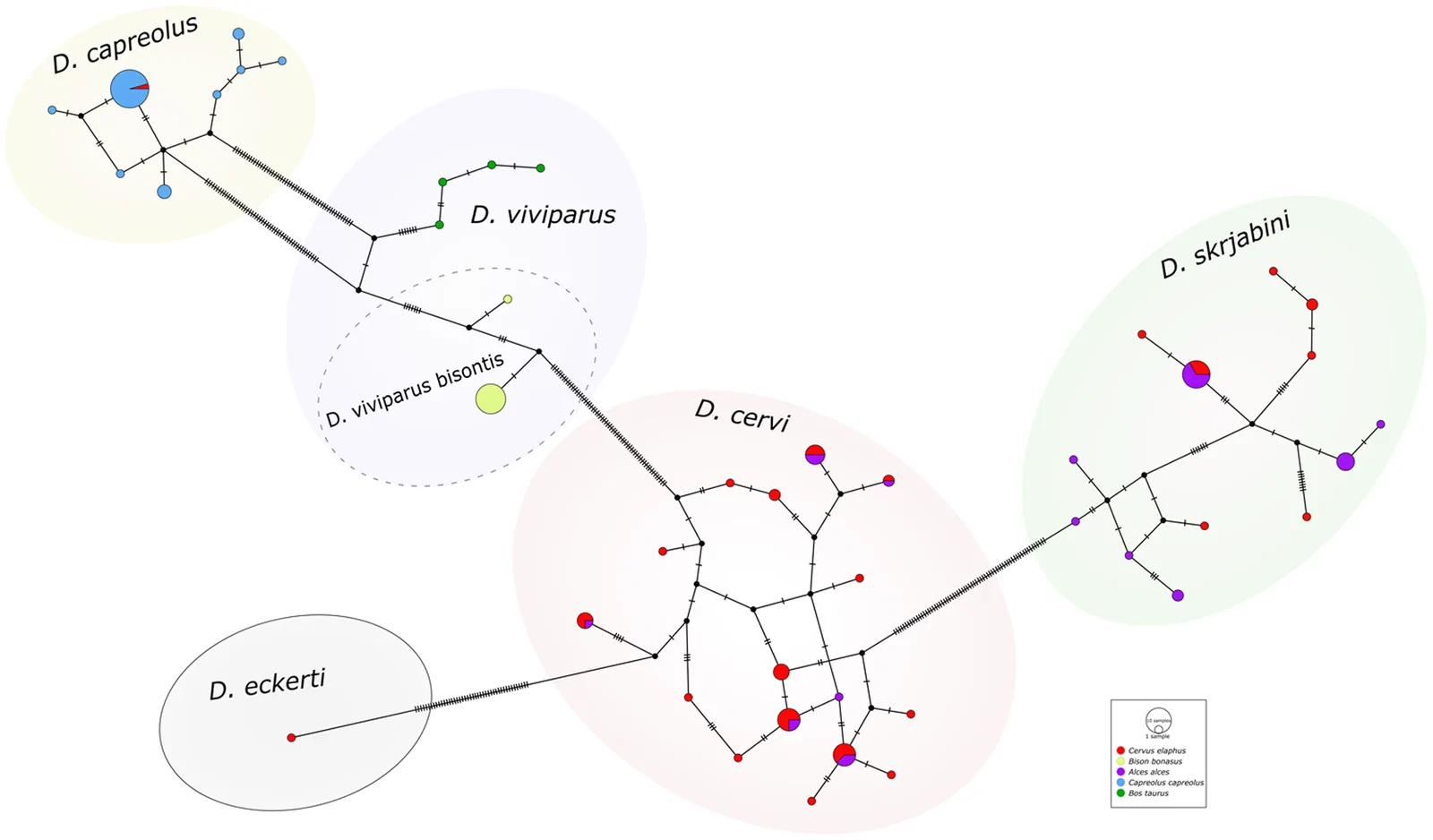
Statistical parsimony phylogenetic network based on 125 *Dictyocaulus* spp. *Cox1* sequences (592 bp) obtained in this study and retrieved from GenBank. Each circle represents a unique haplotype, with circle size proportional to its frequency. Circle colours indicate host species. Hatch marks along connecting lines denote the number of mutational steps, and black dots represent missing (unsampled) intermediate haplotypes. Figure 7 long description.

The TCS haplotype network based on *cox1* sequences was constructed using sequences generated in this study together with reference sequences obtained from GenBank (Supplement Table 2). The analysis revealed 5 clearly separated *Dictyocaulus* clusters, with no shared haplotypes between taxa (Figure 7). Four of these clusters corresponded to species identified in the analysed samples: *D. capreolus, D. cervi, D. skrjabini* and *D. viviparus bisontis. Dictyocaulus skrjabini* formed a distinct, well-supported cluster composed of several closely related haplotypes, primarily detected in moose. *Dictyocaulus cervi* exhibited the highest haplotype diversity, forming a dense network of interconnected haplotypes largely associated with red deer. *Dictyocaulus capreolus* also formed a separate cluster characterized by moderate diversity, with haplotypes mainly originating from roe deer. The *D. viviparus* group consisted of 2 well-defined units: a compact cluster corresponding to *D. viviparus bisontis* detected in European bison, and a small, clearly separated cluster representing *D. viviparus* from cattle. No intermediate or transitional haplotypes were observed between *D. viviparus bisontis* and *D. viviparus*, supporting their genetic distinctiveness. Several sequences previously identified as *D. eckerti* clustered within the *D. cervi* haplotype network. In contrast, a single *D. eckerti* haplotype originating from New Zealand formed a distinct and well-separated cluster. Reference sequences from Hungary were also grouped within the *D. cervi* cluster. A separate TCS haplotype network based on *SSU rDNA* sequences was also constructed (Supplementary Fig. 2, Supplement Table 3). In comparison with the *cox1* dataset, the *SSU* sequences showed markedly lower variability, with only minimal nucleotide differences among most haplotypes. As a result, the *SSU*-based network provided limited resolution for distinguishing closely related *Dictyocaulus* taxa. In particular, D. *viviparus* and D. *viviparus bisontis* were not clearly separated.

**Figure 8.**
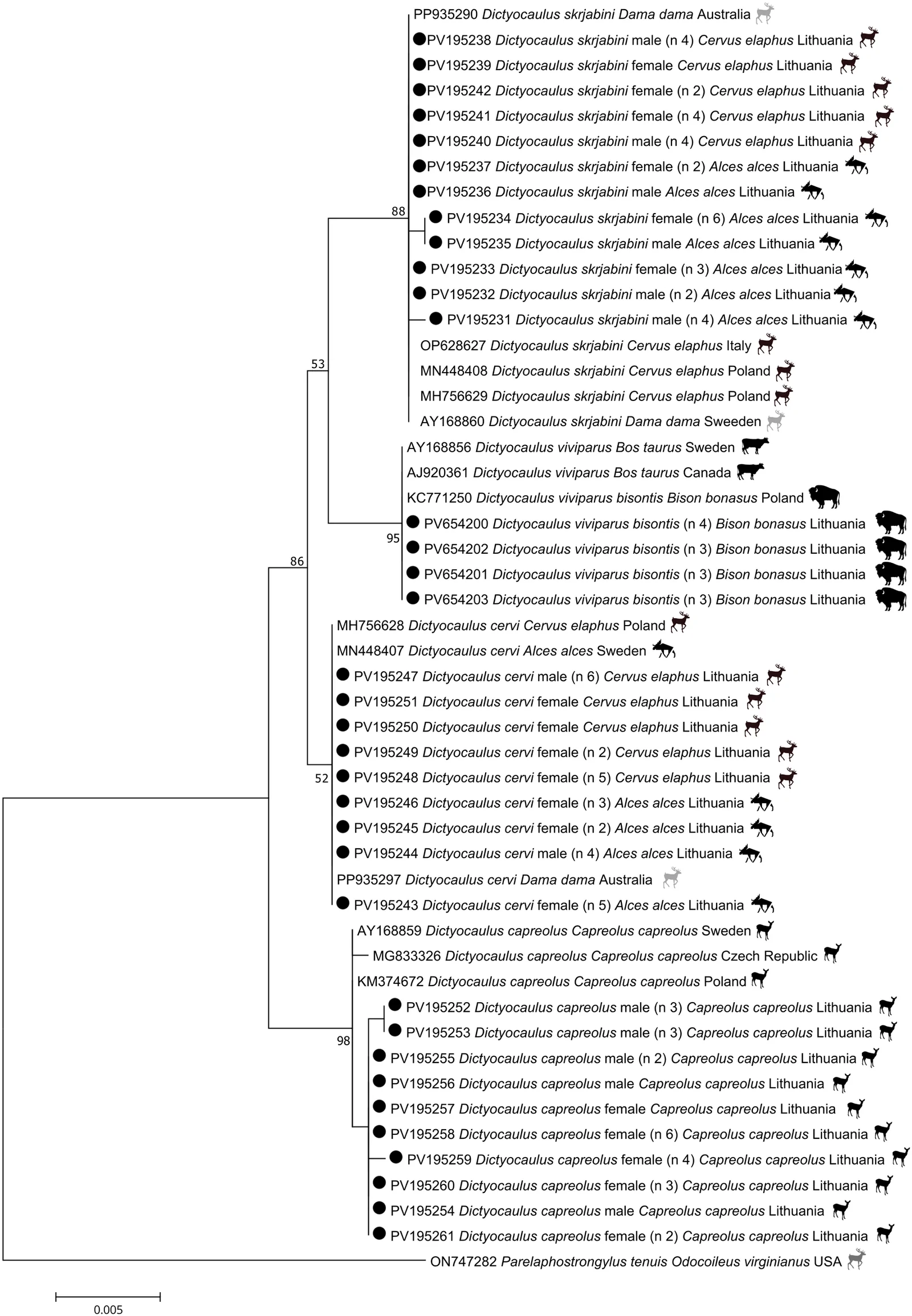
Phylogenetic analysis using the Maximum Likelihood method based on *SSU rDNA* gene sequences (1355 bp) of *Dictyocaulus* spp. From ungulate lungworms. Sequences obtained in this study are marked with ●. Figure 8 long description.

A total of 101 *cox1* sequences and 102 *SSU rDNA* sequences were obtained and analysed. Phylogenetic analysis of *cox1* sequences revealed 4 well-supported clades corresponding to *D. capreolus, D. cervi, D. skrjabini* and *D. viviparus bisontis* (Supplementary Fig. 3). A similar clustering pattern was observed in the *SSU rDNA* phylogenetic tree (Figure 8).

Analysis of *cox1* sequences identified 130 variable positions. The highest genetic diversity was observed in *D. cervi*, which exhibited 12 haplotypes, whereas *D. skrjabini, D. capreolus* and *D. viviparus bisontis* showed 9, 3 and 3 haplotypes, respectively.

The *SSU rDNA* sequences showed lower variability, with 22 variable positions. Correspondingly, lower haplotype diversity was observed, with a single haplotype detected for *D. cervi* and *D. viviparus bisontis* and 2 and 3 haplotypes for *D. skrjabini* and *D. capreolus*, respectively.

Comparison of the phylogenetic trees derived from the mitochondrial *cox1* and nuclear *SSU rDNA* revealed differences in the topology of *Dictyocaulus* species. In the *cox1*-based tree, *D. viviparus* and *D. capreolus* were placed as neighbouring clades, whereas *D. skrjabini* and *D. cervi* formed separate species-level clades. In contrast, the *SSU rDNA* tree showed a different arrangement, in which *D. skrjabini* was placed closest to *D. viviparus*, while *D. capreolus* and *D. cervi* formed separate lineages.

## Discussion

This study presents the first report of *Dictyocaulus* spp. infection in free-ranging wild ungulates from Lithuania. Molecular analysis confirmed the presence of 4 species: *D. skrjabini, D. capreolus, D. cervi* and *D. viviparus bisontis*. The prevalence of *Dictyocaulus* infections varied among host species and according to the nematode species identified. Mixed infection patterns were uncovered, with single *Dictyocaulus* species found in roe deer and European bison and co-infections in red deer and moose. The highest parasite diversity and the greatest degree of aggregation were observed in red deer, highlighting their potential role as a key host in the maintenance and transmission of *Dictyocaulus* spp. in the study area.

*Dictyocaulus skrjabini* is a recently described nematode species. It was first identified in red deer in Poland and subsequently reported in both fallow deer (*Dama dama*) and red deer in Sweden (Pyziel et al., 2023). The species has also been recorded in fallow deer in Australia (Brown et al., 2024) and in red deer in Hungary, where it was previously identified as *Dictyocaulus* sp. (Ács et al., 2016). In the present study, *D. skrjabini* was detected in both red deer and moose. Based on the available scientific literature, this represents the first confirmed record of this species in moose. Its occurrence in multiple cervid hosts suggests a broader host range than initially assumed, particularly in regions where host species coexist. The confirmation of *D. skrjabini* in Lithuanian red deer further supports its widespread distribution across Europe.

*Dictyocaulus capreolus* is a lungworm species originally described in roe deer and moose in Sweden (Gibbons and Höglund, 2002). Since then, it has been widely reported in roe deer populations across Europe, including Spain, Ukraine, Turkey, Hungary, Poland, the Czech Republic and Germany (Carreno et al., 2009; Kuzmina et al., 2010; Bolukbas et al., 2012; Ács et al., 2016; Pyziel et al., 2017; Jurankova et al., 2019; Wild et al., 2025). In the present study, *D. capreolus* was detected predominantly in roe deer, but also occurred in red deer. Previous studies have also documented this species in other hosts, including moose in Sweden (Divina et al., 2002), chamois (*Rupicapra rupicapra*) in Spain (Carreno et al., 2009) and red deer in Hungary (Ács et al., 2016). Although *D. capreolus* is generally considered to be closely associated with roe deer, these findings support the view that the species is capable of infecting a broader range of ungulate hosts, particularly in areas where different host species occur sympatrically (Pyziel et al., 2023). Nevertheless, its predominance in roe deer in the present study is consistent with a high degree of host specificity.

*Dictyocaulus cervi* was first described in red deer population in Poland (Pyziel et al., 2017). Subsequent studies have reported this species in moose in Poland (Filip-Hutsch et al., 2020), in fallow deer in Australia (Brown et al., 2024) and again in red deer in Spain (González-Velo et al., 2025). In addition, *D. cervi*-like lungworms have been identified in rocky mountain elk (*Cervus canadensis nelsoni*) in the USA (Bangoura et al., 2021). Prior to its formal description, *D. cervi* was frequently misidentified as *D. eckerti* in cervid hosts, reflecting the morphological similarity between these taxa and the limitations of traditional identification methods (Ács et al., 2016). In the present study, *D. cervi* was confirmed in both red deer and moose in Lithuania, consistent with previous observations. Notably, this species was the dominant lungworm in red deer, accounting for the majority of recovered specimens. Its high prevalence and abundance suggest that *D. cervi* plays a central role in shaping lungworm infection dynamics in cervid populations in Lithuania. The occurrence of this species in multiple host taxa further indicates a relatively broad host range compared with other *Dictyocaulus* species.

*Dictyocaulus viviparus* is the most widely distributed lungworm species infecting domestic and wild bovids (Ploeger, 2002). A distinct subspecies, *D. viviparus bisontis*, was originally described in the European bison population in Poland based on morphological characteristics, molecular analysis and host specificity (Pyziel et al., 2020). In the present study, this taxon was confirmed in European bison in Lithuania, further supporting its occurrence in free-ranging bison populations in Northern Europe. Notably, despite the relatively low prevalence observed in this host species, infected individuals harboured comparatively high parasite burdens. This pattern may reflect limited exposure but increased susceptibility, potentially associated with host population structure or restricted gene flow. The apparent host specificity of this taxon also suggests a well-adapted parasite–host relationship within European bison populations (Pyziel et al., 2020). A comparable host-associated pattern has been reported in North American bison (*Bison bison*), which harbour a genetically distinct and currently undescribed *Dictyocaulus* species. This taxon differs from both cattle-associated *D. viviparus* and *D. viviparus bisontis* described in European bison, suggesting independent parasite–host associations within the 2 bison species (Danks et al., 2022).

In this study, co-infections involving *Dictyocaulus* spp. were observed in both moose and red deer, with mixed infections of *D. skrjabini* and *D. cervi* occurring in both host species, and occasional involvement of *D. capreolus* in red deer. Previous studies have reported co-infections of *D. cervi* with protostrongylid lung nematodes in moose, which were associated with more severe parasitic bronchitis and pulmonary pathology (Filip-Hutsch et al., 2020). Experimental cross-transmission studies have further demonstrated that both red deer and cattle can be infected with *D. viviparus* and *D. eckerti* (Johnson et al., 2003), although host-specific differences in establishment success were observed. In particular, *D. eckerti* appears to infect cervids more successfully, whereas *D. viviparus* establishes more efficiently in cattle. Together, these findings suggest that co-infections and cross-species transmission may play an important role in shaping lungworm community structure in wild ungulates, particularly in ecosystems where multiple host species coexist.

In the present study, mitochondrial (*cox1*) and nuclear (*SSU rDNA*) markers reliably resolved all 4 *Dictyocaulus* species, providing strong molecular support for their distinct taxonomic status. Given the close morphological similarity among *Dictyocaulus* species, morphology alone is insufficient for accurate species identification, highlighting the necessity of molecular confirmation. Sequences of both markers clearly differentiated *D. skrjabini, D. capreolus, D. cervi* and *D. viviparus bisontis*, with no evidence of overlapping or intermediate genotypes among taxa. These findings are consistent with previous studies demonstrating that molecular markers enable reliable discrimination of closely related *Dictyocaulus* species (Divina et al., 2000, 2002; Höglund et al., 2003; Ács et al., 2016; Pyziel et al., 2017, 2023; Cafiso et al., 2023), and confirm the value of these markers as essential tools for accurate species identification in epidemiological studies.

Although both *cox1* and *SSU rDNA* enabled molecular identification of the analysed *Dictyocaulus* species, the 2 markers differed in their ability to resolve phylogenetic relationships among taxa. The mitochondrial *cox1* marker showed greater sequence variability and provided a more resolved phylogenetic structure, whereas *SSU rDNA* was considerably more conserved. Consequently, some internal relationships in the *SSU rDNA* tree received low bootstrap support, particularly the grouping of *D. viviparus* and *D. skrjabini,* which was supported by a bootstrap value of only 53%. However, this value should be interpreted in the context of the limited variability of the *SSU rDNA* marker. The low bootstrap support does not necessarily indicate weak molecular differentiation between these species but rather may reflect the small number of phylogenetically informative sites available in the *SSU rDNA* dataset. Thus, while *SSU rDNA* allows these species to be distinguished at the molecular level, it provides limited resolution for inferring their relative evolutionary proximity. Therefore, the observed topological differences between the mitochondrial and nuclear phylogenies are more likely to reflect differences in marker variability and phylogenetic signal than genuine discordance between mitochondrial and nuclear evolutionary histories. Further studies incorporating multiple independent mitochondrial and nuclear loci, analysed both separately and in concatenated datasets, would provide a more robust framework for resolving evolutionary relationships within *Dictyocaulus*, as demonstrated in multilocus studies of other nematode taxa (Lefoulon et al., 2017; Kulpa et al., 2025).

Furthermore, the interpretation of GenBank sequences labelled as *D. eckerti* requires caution. Some of these sequences were deposited before the recent taxonomic revision of the genus, and therefore their original species assignment may not fully correspond to the present taxonomy. Consequently, sequences annotated as *D. eckerti* were not considered definitive evidence of a separate *D. eckerti* lineage in the present analysis. This highlights the importance of critically evaluating public database annotations when interpreting phylogenetic relationships.

Phylogenetic analyses based on *cox1* and *SSU rDNA* revealed complex evolutionary relationships among the examined *Dictyocaulus* species. Discrepancies between mitochondrial and nuclear markers have also been reported in other animal groups (Li et al., 2016; Kumar et al., 2017), and are often attributed to processes such as hybridization, introgression or incomplete lineage sorting. In the present study, co-infections with multiple *Dictyocaulus* species within the same host were relatively common, potentially creating opportunities for such processes to occur. Although direct evidence for hybridization or gene flow was not observed, the detected phylogenetic patterns may be consistent with these mechanisms. Further studies, including population-level genetic analyses, are required to clarify the evolutionary relationships and potential genetic exchange among *Dictyocaulus* species.

The true prevalence of *Dictyocaulus* infections is likely underestimated, as assessments often rely solely on the detection of adult nematodes in the bronchi and bronchioles. This limitation underscores the need for the integration of molecular diagnostic approaches and more systematic surveillance across host species and geographic regions. Further studies are required to clarify the epidemiology, clinical significance and ecological interactions associated with co-infections involving different *Dictyocaulus* species, particularly in wildlife populations where multiple taxa may circulate simultaneously.

## Conclusions

This study provides the first comprehensive report of *Dictyocaulus* spp. infections in free-ranging wild ruminants in Lithuania, combining morphological and molecular approaches. Four species (*D. skrjabini, D. capreolus, D. cervi* and *D. viviparus bisontis*) were identified, revealing clear differences in prevalence, host specificity and infection patterns among host species. Red deer were identified as the principal host, exhibiting the highest prevalence, parasite diversity and mean abundance, whereas European bison showed low prevalence but relatively high infection intensity. The detection of co-infections and the occurrence of multiple *Dictyocaulus* species within the same host highlight the complexity of lungworm transmission dynamics in wildlife. The application of molecular markers proved essential for accurate species identification, particularly given the morphological similarity among taxa. These findings contribute to a better understanding of the epidemiology and host–parasite relationships of *Dictyocaulus* spp. and emphasize the need for integrated surveillance approaches in wildlife populations.

## Supporting information

10.1017/S0031182026102704.sm001 Misailovė-Ribokė et al. supplementary material Misailovė-Ribokė et al. supplementary material

## Supplementary material

The supplementary material for this article can be found at https://doi.org/10.1017/S0031182026102704.

## Table 1 Long description

Columns list target gene, primer, oligonucleotide sequences from five prime to three prime, and reference. For target gene, cox1, primer, lco1490 has sequence, five prime ggtcaacaaatcataaagatattgg three prime, with reference, folmer et al., 1994. Under the same target gene, primer, hco2198 has sequence, five prime taaacttcagggtgaccaaaaaatca three prime, with no reference shown in its row. For target gene, ssu rdna, primer, nf50 has sequence, five prime tgaaatgggaacggctcat three prime, with reference, pyziel et al., 2017. Under the same target gene, primer, bnr1 has sequence, five prime acctacagataccttgttacgac three prime, with no reference shown in its row.

Navigate back to Table 1.

## Figure 7 Long description

The diagram is a statistical parsimony phylogenetic network based on 125 sequences. It features several clusters, each represented by circles of varying sizes, indicating unique haplotypes. The size of each circle is proportional to its frequency and different colors represent different host species. The clusters are labeled as D. capreolus, D. viviparus, D. viviparus bisontis, D. cervi, D. skrjabini and D. eckerti. Hatch marks along the connecting lines denote the number of mutational steps, while black dots represent missing intermediate haplotypes. The D. capreolus cluster is shown in blue, D. viviparus in green, D. viviparus bisontis in yellow, D. cervi in red, D. skrjabini in purple and D. eckerti in grey. Each cluster is visually separated by shaded areas, indicating distinct groups with no shared haplotypes between them.

Navigate back to Figure 7.

## Figure 8 Long description

The phylogenetic tree is constructed using the Maximum Likelihood method based on gene sequences of Dictyocaulus species. The tree is oriented vertically, with branches extending from a common ancestor at the bottom. Each branch represents a different sequence, labeled with identifiers and host information. The tree includes sequences from various hosts such as Cervus elaphus, Alces alces and Bos taurus, among others. The tree is divided into several clades, with bootstrap values indicated at the nodes, such as 88, 53 and 85, representing the confidence of the branching. Sequences obtained in this study are marked with a black circle. The tree shows distinct clades for D. skrjabini, D. viviparus bisontis and D. cervi, with sequences from different geographical locations like Lithuania, Poland and Sweden. The scale bar at the bottom represents genetic distance.

Navigate back to Figure 8.

## Table 2 Long description

Host species are listed with the number studied, the number infected, Dictyocaulus spp. identified, prevalence with a 95% confidence interval, mean abundance, and infection intensity range. For C. elaphus, N 41 and n 27, D. skrjabini has prevalence 29.3% with 95% confidence interval 17.6 to 44.5, mean abundance 2.73, and intensity 1 to 45; D. capreolus has prevalence 2.4% with 95% confidence interval 0.4 to 12.6, mean abundance 0.27, and intensity 11 to 11. For C. capreolus, N 33 and n 7, D. capreolus has prevalence 21.2% with 95% confidence interval 10.5 to 37.0, mean abundance 0.88, and intensity 1 to 9. For A. alces, N 14 and n 5, D. cervi has prevalence 35.7% with 95% confidence interval 16.3 to 61.2, mean abundance 1.79, and intensity 1 to 10. For A. alces, D. skrjabini has N 6, prevalence 42.9% with 95% confidence interval 21.4 to 67.4, mean abundance 1.71, and intensity 2 to 11. For B. bonasus, N 41 and n 5, D. viviparus bisontis has prevalence 12.2% with 95% confidence interval 5.3 to 25.3, mean abundance 2.51, and intensity 5 to 57.

Navigate back to Table 2.

## Table 3 Long description

Counts of lungworms are listed by host species and parasite species, split by male and female worms, plus a column for hosts with co-infection per number of infected hosts. For red deer, D. capreolus counts were 4 males and 7 females; D. cervi counts were 321 males and 591 females; D. skrjabini counts were 54 males and 58 females; D. viviparus bisontis was shown as a dash for males and blank for females. Red deer co-infections were 6 of 32 for D. cervi plus D. skrjabini, and 1 of 32 for D. capreolus plus D. cervi plus D. skrjabini with an asterisk marker. For roe deer, D. capreolus counts were 10 males and 19 females, while D. cervi, D. skrjabini, D. viviparus bisontis, and both co-infection columns were shown as dashes. For moose, D. cervi counts were 7 males and 18 females and D. skrjabini counts were 9 males and 15 females, with D. capreolus and D. viviparus bisontis shown as dashes or blanks. Moose co-infection for D. cervi plus D. skrjabini was 4 of 8, and the three species co-infection column was a dash. For European bison, only D. viviparus bisontis had counts, 37 males and 66 females, and all other parasite and co-infection entries were shown as dashes or blanks.

Navigate back to Table 3.
